# Chagas Disease across the Ages: A Historical View and Commentary on Navigating Future Challenges

**DOI:** 10.3390/microorganisms12061153

**Published:** 2024-06-06

**Authors:** Denis Sereno, Bruno Oury, Mario J. Grijalva

**Affiliations:** 1 INTERTRYP, University Montpellier, Centre International de Recherche en Agronomie (CIRAD), Institut de Recherche pour le Développement (IRD), GloInsect: Global Infectiology and Entomology Research Group, 34032 Montpellier, France; bruno.oury@ird.fr; 2 Infectious and Tropical Disease Institute, Biomedical Sciences Department, Heritage College of Osteopathic Medicine, Athens, OH 45701, USA; grijalva@ohio.edu

**Keywords:** Chagas disease, *Trypanosoma cruzi*, vector control, prevention, access to diagnosis, treatment, history, multidisciplinary

## Abstract

Chagas disease, discovered over a century ago, continues to pose a global health challenge, affecting millions mainly in Latin America. This historical review with commentary outlines the disease’s discovery, its evolution into a global concern due to migration, and highlights significant advances in diagnostics and treatment strategies. Despite these advancements, the paper discusses ongoing challenges in eradication, including vector control, congenital transmission, the disease’s asymptomatic nature, and socioeconomic barriers to effective management. It calls for a multidisciplinary approach, enhanced diagnostics, improved treatment accessibility, and sustained vector control efforts. The review emphasizes the importance of global collaboration and increased funding to reduce Chagas disease’s impact.

## 1. Introduction

Chagas disease (CD), also known as American trypanosomiasis, is a tropical parasitic disease that poses a significant health threat to millions of people, primarily in Latin America. It was discovered by the Brazilian physician Carlos Chagas in 1909 [1]. However, evidence of the disease has been identified in pre-Columbian mummies [2], indicating its presence in the Americas long before European contact. This disease involves complex dynamics between humans, vectors (insects from the Triatominae subfamily, commonly known as “kissing bugs”), and both domestic and wild animals. Understanding the historical context of CD is crucial for grasping its current impact and the ongoing challenges in its management and control. This article traces key research milestones from the discovery of CD to the present day, examines the evolution of strategies for treatment and vector control, and highlights the challenges faced in controlling its spread.

## 2. The Discovery and Early Research

The story of CD begins with Carlos Chagas, who first identified the causative agent of the disease in 1909 in Brazil. Dr. Carlos Chagas was assigned the significant task of organizing a public health campaign aimed at combating malaria. In the course of his research, he made several groundbreaking discoveries. Initially, Dr Chagas identified a new type of flagellate parasite in the rectal contents of a triatomine insect, a type of bug commonly found in rural areas of Latin America. His research then led him to detect the same parasite in the blood of a cat, suggesting the possibility of transmission between animals and insects. Most importantly, he later found this parasite in the blood of a young girl, confirming the potential for human infection. This series of discoveries was pivotal, not only in understanding the transmission cycle of what would later be known as CD but also in highlighting the complex interactions between various hosts and vectors in the spread of parasitic diseases [3]. This discovery was groundbreaking as it marked the first time a disease was found to be caused by a parasite transmitted by an insect, a triatome, in the western hemisphere. Chagas’ work extended beyond identifying the pathogen; he described the life cycle of *T. cruzi*, host–parasite interactions, and clinical manifestations, laying the foundation for understanding of the disease and future research [4,5].

Despite Carlos Chagas’ comprehensive and groundbreaking work, CD did not initially receive the attention it deserved. This oversight can be partly attributed to the socio-political context of Brazil at the time, which may have influenced priorities in public health and research funding. Additionally, the complex nature of the disease itself, involving a cycle of transmission that includes vectors, humans, and various animals, made it difficult to address effectively. It would take several decades before the global health community recognized the significance of CD. Only then did international organizations and health authorities begin concerted efforts to study, prevent, and treat it. These efforts included advancing diagnostic techniques, developing treatment protocols, and implementing vector control measures. The delayed response highlights the challenges of raising awareness and mobilizing resources for diseases that primarily affect marginalized populations.

Originally thought to be confined to the rural areas of Latin America, this trypanosomiasis has now been identified in many countries across the globe (https://www.who.int/publications/i/item/20110630-JPN accessed on 2 June 2024). An estimated 288,000 people infected by *T. cruzi* currently live in the United States. This includes 57,000 patients with CD cardiomyopathy and 43,000 women of reproductive age who are infected. Annually, there are between 22 and 108 congenital infections. Additionally, about 10,000 prevalent cases of locally acquired *T. cruzi* infection occur in the United States. Migrations and travel have led to reports of it in 19 non-endemic countries outside Latin America, including Canada and the United States of America, and in many European and some African, Eastern Mediterranean and Western Pacific countries like Japan and Australia, highlighting the disease’s footprint beyond Latin America (https://www.who.int/publications/i/item/20110630-JPN accessed on 2 June 2024). The spread of the disease has been facilitated by migration and the subtle nature of its early symptoms, leading to underdiagnosis and underreporting.

According to the WHO, the public health impact of CD is significant, with an estimation of 6 to 7 million people infected worldwide (https://www.who.int/health-topics/chagas-disease#tab=tab_1, accessed on 2 June 2024). The disease evolves in two phases, acute and chronic. The acute phase often goes unnoticed due to its mild symptoms, but the chronic phase can lead to severe heart, digestive and even urinary disorders, causing long-term disability and death. The economic and social burden of CD is immense, particularly in endemic regions where access to healthcare is limited and awareness of the disease is low.

## 3. Advances in Diagnostics and Treatment

Over the years, research into CD has achieved several milestones. Early methods for diagnosing the disease, such as xenodiagnoses, were developed by Brumpt in 1914 [6]. The development of serological tests in the 1970s [7,8] greatly improved the diagnosis of the disease, facilitating earlier detection and treatment. Currently, the World Health Organization recommends employing two conventional tests that rely on different principles and detect different antigens [9]. In cases where results are ambiguous or discordant, a third technique, such as Western blot [10] or Polymerase Chain Reaction [11], should be utilized. However, these techniques require sophisticated laboratories, unavailable in the primary or secondary healthcare facilities of endemic countries. Moreover, Polymerase Chain Reaction’s sensitivity is low in the chronic phase of the disease when the circulating parasites are rare, and few confirmatory tests based on the Western blot method are globally available. Advancements in diagnostic technology have led to the validation of a loop-mediated isothermal amplification assay (*T. cruzi*-LAMP) for early-stage infections and the local endorsement of various quick diagnostic tests (Rapid diagnostic tests, RDTs) for long-term infections, making testing easier in areas with limited resources [12,13]; the interest of low invasiveness biological sampling should also be further explored [14]. In addition, efforts have been made to improve the screening of blood donations in endemic regions to prevent transfusion-transmitted CD.

The introduction of benznidazole in 1966 [15] and nifurtimox in 1970 [16], two drugs effective in treating the acute phase, marked a significant advancement in management strategies. However, these treatments are not without challenges [17], as they are less effective in the chronic phase and can have severe side effects. Shorter treatment protocols [18], simplified diagnostic algorithms [12], and pediatric formulations of existing drugs [19] will facilitate treatment. In addition, the development of drug resistance, in conjunction with the fact that Nifurtimox and Benznidazole are considered as “obsolete” urge the development of new alternative [20]. New therapeutic options involving new or repurposed drugs [21,22,23] are urgently needed. Fexinidazole is undergoing clinical trials for the treatment of (NCT03587766), and the oxaborole DNDI-6148 has been nominated as a clinical candidate for the treatment of Chagas disease and is in phase I clinical trials (EudraCT 2018-004023-37) [24]. However, post-therapeutic cure still stands as a major challenge since etiological cure is difficult to ascertain [25,26].

The efforts to develop a vaccine has been hampered by the complex life cycle of the *T. cruzi* parasite and the diverse clinical manifestations of the disease. For CD, the vaccine needs to be safe and economically attractive, especially in the world’s low- and middle-income countries [27]. Therapeutic vaccines represent an attractive opportunity with potential positive return on investment [28], and new therapeutic vaccines or immunotherapies are under development using multiple platforms, including IVT mRNA [27,29]. Finally, to improve transmission prevention, a better understanding of the genetic diversity of *T. cruzi* [30] and an improvement in vector control strategies [31] are required. 

## 4. Challenges in Eradication and Control

The primary triatomine vectors of CD belong to the Hemiptera order, Heteroptera suborder, Reduviidae family, Triatominae subfamily. The biodiversity of triatomine insects is extensive, with 157 validated species (154 extend and three extinct) and 2 subspecies, distributed in 18 genera and five tribes: Alberproseniini, Bolboderini, Cavernicolini, Rhodniini, and Triatomini. Most of the diversity (143 species) is restricted to Latin America [32,33] and Asia (14 species). Notable among the over 100 Triatoma species identified as proven *T. cruzi* vectors are *Triatoma infestans*, predominant in South America’s rural Southern Cone; *Rhodnius prolixus*, significant in Northern South America; *Triatoma dimidiata* with a wide distribution across Mexico, Central America, and Northern South America; *Rhodnious ecuadoriensis* present in Ecuador and Northern Peru; and *Panstrongylus megistus*, mainly found in Brazil.

Despite progress, controlling CD remains a formidable challenge. The adaptability of these vectors to both wild and domestic settings, particularly in substandard housing conditions, presents a challenge to disease control efforts [34]. Addressing this issue necessitates a comprehensive strategy, encompassing insecticide application, structural improvements to housing [35], and extensive community education to mitigate the spread of the disease effectively. Additionally, the asymptomatic nature of the disease’s early stage, the high genetic diversity of *T. cruzi*, and the lack of a vaccine make prevention difficult. In addition, the mobility of populations from endemic to non-endemic regions continue to challenge blood safety protocols worldwide [36]. Furthermore, the risk of transmission from mother to child is estimated to be 1 to 5%, depending on the mother’s clinical status and the region [37]. Infected newborns may be asymptomatic or present symptoms such as low birth weight, anemia, or hepatosplenomegaly. As such, screening for Chagas disease is recommended for pregnant women in endemic areas to enable timely intervention and reduce congenital transmission rates. The detection and prompt treatment of congenital cases of Chagas disease is essential for long-term elimination of transmission. 

Social and economic factors, such as poverty and limited access to healthcare in endemic regions, exacerbate the challenges of addressing Chagas disease, making it a complex issue that requires a multidisciplinary approach for effective management. Recognizing the severity of these challenges, the World Health Organization (WHO) has emphasized the need for comprehensive and equitable access to healthcare and services for everyone affected by CD. In response to this, WHO has issued a call to action, urging member states to implement strategies that ensure all patients can receive the necessary care, particularly in underserved areas. This approach aims not only to treat the disease but also to improve the quality of life for those affected, breaking the cycle of transmission and reducing the disease’s impact on vulnerable populations (https://www.who.int/news/item/14-04-2021-who-calls-for-comprehensive-equitable-access-to-healthcare-for-every-chagas-disease-patient, accessed on 2 June 2024).

## 5. Current Status and Future Directions 

Today, Chagas disease remains a significant public health concern. Research is ongoing in several critical areas, including the development of more effective treatments and diagnostic tests that can be used at the point of care. Additionally, efforts are being made to develop vaccines and improve vector control strategies. There has been an increase in international collaboration and funding, underscoring the global recognition of CD as a health priority. Looking ahead, it is essential to focus on enhancing diagnostic capabilities and the accessibility of treatments. Public awareness must also be raised to better understand the disease’s transmission and impact. Sustained vector control programs are crucial; these should include comprehensive entomological surveys of triatomine vectors and the application of insecticides to infested dwellings. Furthermore, future strategies to mitigate the risk of *T. cruzi* transmission through congenital means, blood donations, and organ transplants must prioritize global collaboration. This includes adopting universal screening methods and developing more sensitive, cost-effective diagnostic tools to prevent the spread of this debilitating disease.

## Data Availability

Not applicable.
